# Validation into Spanish of a Scale to Detect the Post-intensive Care Syndrome*

**DOI:** 10.17533/udea.iee.v41n1e09

**Published:** 2023-03-14

**Authors:** Mario Andrés Narváez Martínez, Ángela María Henao Castaño

**Affiliations:** 1 Nurse, Master’s. Fundación Cardioinfantil - Instituto de Cardiología, Bogotá Colombia. Universidad Nacional de Colombia; Bogotá Colombia. E-mail: manarvaezma@unal.edu.co. Universidad Nacional de Colombia Universidad Nacional de Colombia Bogotá Colombia manarvaezma@unal.edu.co; 2 Nurse, PhD. Universidad Nacional de Colombia; Bogotá Colombia. E-mail: angmhenaocas@unal.edu.co Universidad Nacional de Colombia Universidad Nacional de Colombia Bogotá Colombia angmhenaocas@unal.edu.co

**Keywords:** sobrevivientes, cuidados críticos, síndrome, cuestionario de salud del paciente, psicometría, Sobreviventes, cuidados críticos, syndrome, questionário de saúde do paciente, psicometria

## Abstract

**Objective.:**

This work sought to validate the Spanish version of the scale Healthy Aging Brain-Care Monitor (HABC-M) scale as clinical tool to detect the Post-intensive Care Syndrome.

**Methods.:**

Psychometric study, conducted in adult intensive care units from two high-complexity university hospitals in Colombia. The sample was integrated by 135 survivors of critical diseases with mean age of 55 years. The translation of the HABC-M was carried out through transcultural adaptation, evaluating content, face, and construct validity and determining the scale’s reliability.

**Results.:**

A replica was obtained of the HABC-M scale in its version into Spanish, semantically and conceptually equivalent to the original version. The construct was determined through confirmatory factor analysis (CFA), evidencing a three-factor model comprised of the subscales: cognitive (6 items), functional (11 items), and psychological (10 items), with a confirmatory factor index (CFI) of 0.99, a Tucker Lewis index (TLI) of 0.98, and an approximate root-mean-square error (RMSE) of 0.073 (90% CI: 0.063 - 0.084). Internal consistency was determined through Cronbach’s alpha coefficient, obtaining 0.94, (95% CI 0.93 - 0.96).

**Conclusion.:**

The Spanish of the HABC-M scale is a tool with adequate psychometric properties, validated and reliable to detect the Post-intensive Care Syndrome.

## Resumen

**Objetivo.:**

Validar la versión en español de la escala Healthy Aging Brain-Care Monitor (HABC-M) como herramienta clínica para la detección del síndrome poscuidado intensivo.

**Métodos.:**

Estudio psicométrico, el cual se llevó a cabo en unidades de cuidado intensivo adulto de dos hospitales universitarios de alta complejidad en Colombia. La muestra fue integrada por un total de 135 sobrevivientes a enfermedades críticas con edad promedio de 55 años. La traducción del HABC-M se realizó mediante adaptación transcultural, evaluándose la validez de contenido, facial, constructo y determinándose la confiabilidad de la escala.

**Resultados.:**

Se obtuvo una réplica de la escala HABC-M en su versión al español, semántica y conceptualmente equivalente a la versión original. El constructo se determinó a través de análisis factorial confirmatorio (AFC), evidenciando un modelo de tres factores compuesto por las subescalas: cognitiva (6 ítems), funcional (11 ítems) y psicológica (10 ítems), con un índice de factores confirmatorios (CFI) de 0.99, un índice de Tucker Lewis (TLI) de 0.98 y una raíz cuadrada del error cuadrático medio aproximado (RMSEA) de 0.073 (IC 90 %: 0.063 - 0.084). La consistencia interna se determinó mediante el coeficiente alfa de Cronbach, obteniendo e 0.94, (IC 95 % 0.93 - 0.96).

**Conclusión.:**

La versión en español de la escala HABC-M es una herramienta con adecuadas propiedades psicométricas, válida y confiable para la detección del síndrome poscuidado intensivo.

## Resumo

**Objetivo.:**

Para validar a versão espanhola da escala Healthy Aging Brain-Care Monitor (HABC-M) como uma ferramenta clínica para a detecção da síndrome do cuidado pós-cuidado.

**Métodos.:**

Estudo psicométrico, que foi realizado em unidades de terapia intensiva para adultos de dois hospitais universitários de alta complexidade na Colômbia. A amostra consistiu em 135 sobreviventes de doenças críticas. A tradução do HABC-M foi realizada por meio de adaptação transcultural, avaliando o conteúdo, facial e construção de validade e determinando a confiabilidade da escala.

**Resultados.:**

Uma réplica da escala HABC-M foi obtida em sua versão em espanhol, semântica e conceitualmente equivalente à versão original. A construção foi determinada através da análise fatorial confirmatória, mostrando um modelo de três fatores composto pelas subescalas: cognitiva (6 itens), funcional (11 itens) e psicológica (10 itens), com um índice fator confirmatório (CFI) de 0.99, um índice de Tucker Lewis (TLI) de 0.98 e um erro médio quadrático aproximado da raiz (RMSEA) de 0.073 (90% CI: 0.063 - 0.084). A consistência interna foi determinada pelo coeficiente alfa do Cronbach, com de 0.94 (95 % CI 0.93 - 0.96).

**Conclusão.:**

A versão espanhola da escala HABC-M é uma ferramenta com propriedades psicométricas adequadas, válida e confiável para a detecção da síndrome do pós-cuidado intensivo.

## Introduction

Intensive care units (ICU), and in them critically ill patients, allow a glimpse into a dynamically uncertain panorama for those who need to enter these; nevertheless, progress in care and the rise of critical diseases keep a close relationship with surviving patients upon hospital discharge.^1^ Survivors of critical diseases are considered fragile individuals due to complications derived from care in ICU, which are related with physical deconditioning, locomotion problems, falls, pain, altered sleep, anxiety, depression, post-traumatic stress disorder, cognitive impairment associated with delirium, mild Alzheimer's, attention deficit, among others.^2^^-^^6^ These complications were defined by the Society of Critical Care Medicine (SCCM) as Post-intensive Care Syndrome (PICS), being a condition that affects between 50% and 70% of patients admitted to ICU.^6^^-^^8^

In 2012, the SCCM defined PICS as one of the new physical, cognitive, and mental health problems related with critical illness and/or its worsening, which persist upon discharge in survivors and involve body, thoughts, feelings, mind and can affect the family.^1^^,^^6^ The PICS is a clinical situation of great social impact, which is manifested rapidly in critical patients and can persist up to five years after the hospital stay; hence, it must be considered an unfavorable situation, which impacts upon the person’s health, family, as well as on the quality of life, return to work and to society.^5^^,^^6^

Globally, an increasing number of critically ill patients survive the ICU stay and - overall - mortality diminishes in the short term.^9^ In the United States, 5.7-million people are admitted to ICU yearly, of which 4.8-million survive;^10^ among the reasons for admission, the acute respiratory distress syndrome (ARDS) affects approximately 200-thousand people per year, among which, nearly 100-thousand survivors must face the complications of the critical illness and its sequelae.^11^ In Colombia, a study on ICU survivors (n = 186) revealed a survival rate of 69% at 38 days; of these, 52.3% of the survivors were unable to resume activities of daily living prior to their admission to ICU.^12^

Currently, PICS detection is complex, given the vast variety of components affected and the lack of a diagnostic test as gold standard. Among the tools used most to assess the physical component, the walk test, the Medical Research Council (MRC) scale, Kats’ index, Barthel’s index, and Lawton’s instrumental activities of daily life are highlighted;^13^^,^^14^^-^^19^ the cognitive component frequently uses the Montreal Cognitive Assessment (MOCA) test, the Repeatable Battery for the Assessment of Neuropsychological Status (RBANS), and the telephone interview for the cognitive status (TICS).^16^^,^^19^^-^^21^ Regarding the mental or psychological component, the hospital anxiety and depression scale (HADS) has been used, as well as Beck’s anxiety inventory, the depression inventory scale, second edition (BDI-II), the post-traumatic stress syndrome (PTSS 14), the event impact scale (EIS), the revised event impact scale (EIS-R), the post-traumatic stress disorder (PTSD) instrument, and the civilian checklist (PCL-C).^13^^,^^15^^,^^16^^,^^22^ Quality of life has also been evaluated through the instruments short-form health survey scale (SF-36), EUROQOL (EQ-5D), and health-related quality of life (HRQOL).^13^^,^^21^^,^^23^^-^^25^

Due to the foregoing and given the use of diverse measurement instruments or scales that assess independently the components affected by PICS, the Healthy Aging Brain-Care Monitor (HABC-M) was validated in the United States. It is a scale composed of 27 items, divided into three subscales, which permits detecting PICS through the cognitive, physical, and psychological dimensions, from the ICU survivor’s perception.^7^ This instrument’s importance for the professional nursing practice lies in contributing to the national Spanish-speaking scientific community with a clinical tool translated, adapted, and validated into Spanish that permits to quickly and simply identify PICS, establish a comprehensive approach and favor the implementation of timely interventions; so that, the rehabilitation and reincorporation of the survivors of critical diseases, in their family, social, and work environment, takes place with the least cognitive, physical, and psychological sequelae, as well as the lowest possible impact on the quality of life. The aim of this study was to validate the Spanish version of the HABC-M scale as a clinical tool to detect PICS.

## Methods

Type of study. Psychometric study, through which the properties of a health measurement scale were evaluated.^26^

Population and sample. The study population were adult patients who had survived a critical illness from two high-complexity university hospitals in Colombia. Sample size was determined for each of the stages in the following manner: (1) in content validity, a panel of 11 experts was formed, highlighting doctors and nurses, specialists in critical care with > 5 years of experience in ICU, who evaluated the HABC-M from criteria of relevance and representativeness;^27^ and (2) for face validity, a panel of judges was integrated made up of 30 survivors of a critical illness with stay in ICU, who provided their concept of the scale in terms of clarity, comprehension, and accuracy; the construct validity was constituted by 135 ICU survivors (*n* = 27 items per 5 participants), exceeding the minimum suggested by Terwee to measure psychometric properties in health questionnaires and following recommendations by diverse authors who propose that, for effects of calculating the sample, for each item there should be between 5 and 10 participants.^27^^-^^29^

For the stages mentioned, a non-probabilistic sampling was carried out for convenience. All patients older than 18 years with mechanical ventilation for ≥ 48 h, and whose discharge from ICU was < 3 months, were consecutively included. The study excluded patients with history of moderate to severe cognitive or neurological disorder, described in the clinical history and of palliative care. The inclusion criterion of mechanical ventilation for the stage of facial validity was not considered, given that this group only evaluated the scale taking into account the aforementioned criteria, thus ending their participation in the study.

Validation phases. For the purposes of this study, the following stages were taken into account: selection of the instrument; translation; validity and reliability tests.^30^

Instrument. The study selected the 27-item HABC-M scale developed in the United States and validated in 2019 by Wang *et al.,* as clinical tool to detect PICS in survivors of critical diseases.^7^ The HABC-M is a scale composed of three subscales: *cognitive*, with 6 questions on memory, orientation and judgment; *functional*, which encompasses 11 questions on instrumental activities and basic activities of daily living (BADL); and *psychological*, with 10 questions on symptoms of depression, psychosis, and anxiety. This instrument lets patients indicate how frequently they experienced symptoms related with PICS during two weeks prior to filling it out. Each question is scored in a scale from zero to three in which the maximum scores of the cognitive, functional, and psychological subscales are 18, 33, and 30, respectively, and the maximum total score is 81, interpreting that, from the high scores for the three subscales and the global scale, there is a higher correlation of the severity of the symptoms associated with PICS.^7^

Translation and adaptation. The HABC-M scale was translated through the transcultural adaptation (TA) technique, which performed a four-phase process.^31^ Phase 1. *Direct translation from English to Spanish:* with participation by three independent bilingual translators, whose native language was the target language. Phase 2. *Synthesis of translations:* a mixed committee (nurse-translator) was in charge of comparing the translations. For this process, the best translated items were selected with the highest idiomatic, semantic, conceptual and cultural equivalence. Phase 3. *Back-translation:* this process was conducted by an official translator, native of the United States, with certification for Spanish-English translation, endorsed by the American Translators Association (ATA). Phase 4. *Conceptual and linguistic equivalence:* It was sought that the general concepts were similar in meaning and fulfilled the same function for all the components of the scale, bearing in mind the population to which it concerns applying the instrument.^32^

Statistical analysis: validity and reliability tests. The content validation stage was based on the modified Lawshe model, with which the level of agreement with the groups was determined, through calculating the content validity rate (CVR) for each item; additionally, the instrument’s global content was calculated through the content validity index (CVI). For this study, CVR ≥ 0.58 and CVI > 0.58 were considered acceptable.^33^ Face validation was conducted from the inter-observer agreement index and measured with Fleiss’ Kappa coefficient, interpreting as satisfactory results categories obtaining values between 0.61 and 0.80, that is, substantial agreement.^34^ Besides the prior statistical analyses, statistical significance tests were applied for both stages. For this, the binomial probability test was used and with which it was sought to determine the percentages of agreements among participants, domains, and the instrument in general. Before performing the statistical analysis of the construct validation stage, a descriptive analysis was conducted of the data provided. Categorical variables were expressed as frequencies (*n*) and percentages (%), and the quantitative variables as measures of central tendency. The construct was determined through confirmatory factor analysis (CFA) with the aid of the structural equations model (SEM) and an estimation through the Weighted Least Square Mean and Variance Adjusted (WLSMVA) method. The final SEM model was interpreted from the following good-fit criteria: confirmatory factor index (CFI) > 0.95, Tucker Lewis index (TLI) > 0.95, and approximate root-mean-square error (RMSE) < 0.08.^12^^-^^15^ Correlations were considered moderate if *r* is between 0.31 and 0.50, and strong if *r* ≥ 0.50 or 1.^35^ The internal consistency of the scale items was evaluated through Cronbach’s alpha coefficient and were considered acceptable values > 0.7 and correlations > 0.31.^35^ Data were analyzed with the STATA/MP statistical program version 14.2 and the Lavaan and Psych R package.

Data collection. The data collection took place from April 2021 to April 2022 by the principal researcher; however, the construct stage had collaboration from nursing professionals, students specializing in critical care from a Colombian university, who were trained through theoretical formation about PICS, knowledge of the HABC-M Instrument, instructions on how to fill out the instrument, and resolution of doubts. During the data collection, approval was obtained from the participants through the informed consent. For the facial and construct validity stages, information collection was carried out in the hospitalization services or in their homes, guaranteeing confidentiality.

Ethical considerations. Authorization to use the instrument was granted by one of the Instrument’s authors, Dr. Boustani. The protocol was approved by the Ethics and Research Committee at Universidad Nacional de Colombia (Endorsement 010-21) and the health institutions where the study was conducted (Act 12-2021 - 007-007 de 2021). All the participants signed the informed consent prior to filling out the scale.

## Results

### Translation and adaptation

The 27 items of the instrument were translated without difficulty; thereafter, the translated and adapted version was presented to one of the authors of the original scale (Dr. Boustani), who validated the TA process carried out, obtaining the first version into Spanish of the HABC-M tool to detect PICS (Annex 1).

### Psychometric properties of the HABC-M Instrument

***Content validation*.** This stage showed, as result, a degree of agreement among experts ≥ 64% (CVR ≥ 0.64) and global CVI in terms of pertinence of 85%, and of 79% in terms of representativeness in all the domains that make up the general scale. Statistical significance tests permitted establishing agreement percentages between 60% and 80% (*p* = 0.01) for the criterion of pertinence, and between 60% and 70% in terms of representativity (*p* < 0.03), with respect to the domains that comprise the instrument and the global scale.

***Face validation*.** The concordance index among ICU survivors, measured through Fleiss’ Kappa coefficient, for the categories of clarity, comprehension, and accuracy of the instrument, showed satisfactory results (Table 1). Statistical significance tests evidenced agreement percentages among survivors, for the three categories, > 80% (*p* < 0.03) in the different subscales and at general level.

**Table 1:** Results of Kappa - HABC-M Instrument

Categories	Global agreement	Free-marginal Kappa
Clarity	0.78	0.71
Comprehension	0.80	0.74
Accuracy	0.80	0.74

*Construct validity.* Regarding the characteristics of the 135 patients surviving a critical illness, it can be noted in Table 2 that the mean age was 55 years ± 15.6 and the predominant gender was masculine (64.4%). Place of origin was most frequently urban (81.5%) and level of schooling outstanding in this population was primary (44.4%), followed by high school (24.4%); and in a similar ratio (15% and 16%) patients with technical and technological levels with respect to survivors with university undergraduate and graduate education. With respect to clinical characteristics, it was noted that the principal motive for admission to ICU was respiratory failure associated with infection due to SARS-CoV-2 (46.7%), followed by other causes (43%). The degree of severity of the illness measured through the Apache II index of admission to ICU was 20.5±7.8. Average days of stay in ICU and of invasive mechanical ventilation were 17±15 and 11±8.9, respectively.

**Table 2:** Characteristics of patients surviving a critical illness (*n*=135)

Sociodemographic variables	Value
Age, mean (SD)	55.18 (15.6)
Masculine, *n* (%)	87 (64.4)
Origin: urban, *n* (%)	110 (81.5)
Schooling, *n* (%)	
Without studies	2 (1.5)
Incomplete primary	37 (27.4)
Complete primary	23 (17)
Incomplete high school	10 (7.4)
Complete high school	20 (14.8)
Technical	15 (11.1)
Technological	6 (4.4)
Undergraduate	15 (11.1)
Graduate	7 (5.2)
Clinical characteristics	
**Conditions of admission to ICU, *n* (%)**	
Respiratory failure associated with infection due to SARS-CoV-2	63 (46.7)
Respiratory failure associated with other causes	19 (14.1)
Other causes	53 (39.3)
Apache II, mean (SD)	20.5 (7.8)
Time in ICU (days), mean (SD)	17 (15)
Time with IMV (days), mean (SD)	11 (8.9)

SARS-CoV-2: Severe Acute Respiratory Syndrome Coronavirus 2; APACHE II: Acute Physiology and Chronic Health Evaluation II; SD: standard deviation; ICU: intensive care unit; IMV: invasive mechanical ventilation

**Figure 1:**
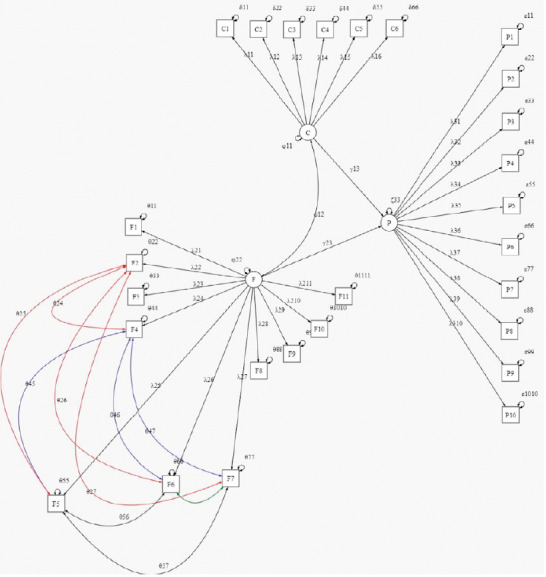
Adjusted structural equations model C: cognitive; F: functional; P: psychological

Confirmatory factor analysis. The CFA yielded a three-factor model (cognitive, functional, and psychological), adjusted from data obtained from ICU survivors. This model demonstrated a fit between good and excellent with an RMSE of 0.073 (90% CI: 0.063 - 0.084), CFI of 0.99, and TLI of 0.98. To determine the adequate fit of the model with respect to the data, the Lavaan *mod-indices* function was used with which additions were made iteratively and with special caution, so that the added parameters were in context of the problem (Figure 1).

The estimated parameters for the SEM were conducted through the marker method, which permitted the first weight of each factor to be fitted into one for comparison purposes (Table 3). The first factor obtained homogenous weights, so that, considering the standard error, they were statistically equal among them and equal to one. The std.all values can be interpreted as correlations (*r*) between the factor and the variable measured with the instrument. Thus, it was possible to evidence that the questions of the questionnaire applied to patients had strong correlation with the values taken by cognitive health from these (r > 0.83). For the second and third factors, diverse weights were obtained; consequently, these factors (functional and psychological) were understood easily through some questions and, to a lesser extent, through others. Both results evidenced strong correlations between the measured variables and the construct (r > 0.56, functional; r > 0.57, psychological). 

**Table 3:** Estimation of weights of fitted SEM

Parameter	Estimation	Standard error	***p* value**	std. all
λ11	1.000	─	─	0.872
λ12	0.995	0.058	<0.0001	0.867
λ13	0.988	0.046	<0.0001	0.862
λ14	0.998	0.057	<0.0001	0.870
λ15	1.021	0.055	<0.0001	0.891
λ16	0.956	0.060	<0.0001	0.834
λ21	1.000	─	─	0.789
λ22	1.086	0.091	<0.0001	0.857
λ23	0.920	0.076	<0.0001	0.726
λ24	0.720	0.093	<0.0001	0.568
λ25	0.730	0.092	<0.0001	0.576
λ26	0.818	0.099	<0.0001	0.645
λ27	0.960	0.079	<0.0001	0.758
λ28	0.879	0.105	<0.0001	0.693
λ29	1.049	0.088	<0.0001	0.828
λ210	1.026	0.090	<0.0001	0.810
λ211	1.029	0.087	<0.0001	0.812
λ31	1.000	─	─	0.826
λ32	0.952	0.061	<0.0001	0.786
λ33	0.953	0.072	<0.0001	0.787
λ34	0.934	0.072	<0.0001	0.771
λ35	1.082	0.089	<0.0001	0.894
λ36	1.050	0.076	<0.0001	0.867
λ37	0.693	0.079	<0.0001	0.573
λ38	0.847	0.062	<0.0001	0.700
λ39	0.990	0.073	<0.0001	0.818
λ310	0.777	0.098	<0.0001	0.642

Std.all: standardized estimation to the entire model.

The CFA under the SEM permitted establishing the regression path of the latent variables (γ) and covariances (*θ*) through their estimations, via statistically significant results according with the estimated parameters (p < 0.001), with correlations for γ between moderate and strong. With respect to the covariances, the results demonstrated that the SEM was adjusted adequately to the data observed, which permitted establishing correlations among the functional health variables (cooking, walking, bathing, shopping, doing household chores), in which body movement is important. These findings allowed to verify that the correlations proposed were significant and interpretable (r > 0.7) (Table 4).

**Table 4:** Estimation of the latent regression parameters

Coefficient	Estimation	Standard error	***p*-valor**	Std.all
γ13	0.329	0.079	<0.0001	0.347
γ23	0.624	0.086	<0.0001	0.596
θ24	0.297	0.066	<0.0001	0.701
θ25	0.344	0.067	<0.0001	0.815
θ26	0.304	0.065	<0.0001	0.772
θ45	0.585	0.079	<0.0001	0.869
θ46	0.475	0.071	<0.0001	0.756
θ47	0.420	0.077	<0.0001	0.782
θ56	0.528	0.072	<0.0001	0.846
θ57	0.467	0.078	<0.0001	0.875
θ67	0.428	0.065	<0.0001	0.858
ϕ12	0.512	0.067	<0.0001	0.744

Std.all: standardized estimation to the entire model.

Reliability. Internal consistency was determined with Cronbach’s alpha coefficient. The HABC-M scale obtained results between good and excellent for the three subscales and the global scale (Cronbach’s alpha of 0.94). The three subscales had correlations between moderate and strong, evidencing that all the items were related with the subscale with no dependence of the scale on a single item (Table 5).

**Table 5:** Reliability of the HABC-M scale

Subscales	Cronbach’s alpha (95% CI)	Correlations (r)
Cognitive	0.90 (0.88 - 0.93)	0.79
Functional	0.90 (0.87 - 0.92)	0.45
Psychological	0.87 (0.84 - 0.90)	0.52
Global scale	0.94 (0.93 - 0.96)	

## Discussion

The HABC-M is a scale with high potential to detect the PICS in survivors of critical diseases, from the alteration of three domains (cognitive, functional, and psychological). The results were consistent and strengthened prior research on the psychometric properties of the HABC-M within the PICS context.^7^^,^^36^ The Spanish version was obtained from the TA process and it was possible to achieve a semantic replica conceptually equivalent to the original version, product of the collaborative work among a philologist, certified translators, methodology experts, and intensive care specialists.

In previous studies, phase and content validity were carried out through expert consensus, evaluating comprehension and clarity criteria in ICU staff.^36^^-^^38^ This study established percentages between experts and survivors in separate samples and defined broader criteria for each of the stages. In previous versions, the construct of the HABC-M instrument was validated through CFA and convergent validity.^7^^,^^36^^-^^38^ The Spanish version was determined through CFA and it was possible to obtain a model with fit indices between good and excellent, which corroborated the studies by Monahan *et al*., on the scale’s adequate psychometric properties through CFA.^37^^,^^38^ Nevertheless, although the RMSE in this study was > 0.05, a figure greater than those found in studies by Monahan *et al*., the literature supported values < 0.08, even up to 0.1, in samples considered small (*n* < 200), which is why it is possible to indicate that the model fitted reasonably the data observed.^39^^-^^41^

Through the estimation of weights, the factorial model allowed establishing strong and significant correlations between the factor and the variable measured with the instrument. This was congruent with previous studies, which tested the scale’s psychometric properties through CFA.^37^^,^^38^ Regarding the regression path of the latent variables under the SEM, it is important to highlight how the functional and psychological subscales correlate with the severity of the PICS symptoms; thus, it was possible to evidence how functional health is causal of damage to psychological health and vice-versa. Regarding cognitive health, the findings suggested that such were less observable, which supported the hypothesis by Wang *et al*., and Horlait *et al*., on the limitations with respect to this subscale and the need to evaluate its objectivity on the severity of the cognitive symptoms in the context of the critical patient;^7^^,^^36^ however, upon the presence of problems in cognitive and/or functional health, it was concluded that a deterioration of these could trigger more severe sequelae in psychological health.

Moreover, significant correlations were found between BADL (cooking, walking, bathing, shopping, and doing household chores) and the ICU stay. This allowed inferring that the difficulty in the body’s mobility is a secondary result in survivors of a critical illness. In this sense, some authors have described how critical patients are prone to experiencing limitations in functional activities that involve body movement and suffering persistent disabilities with BADL affectation is related to PICS.^2^^,^^3^^,^^9^^,^^10^

The results of the present study showed that the HABC-M is a reliable instrument in the PICS context and the findings evidenced in the Spanish version denoted internal consistency between good and excellent, compared with prior studies. The correlations found did not differ from previous studies, which shows the diversity of items among subscales and their importance in the construct.^7^^,^^36^ Due to this, the impact of the present study for the ICU-surviving population, as well as for the nursing discipline, lies on the benefit of having a clinical tool adapted and validated into Spanish, which permits detecting PICS rapidly and simply by applying this scale in clinical or outpatient settings, seeking an integral approach that allows establishing care routes focused on reestablishing cognitive, functional, and psychological health; besides transcending through multidisciplinary and collaborative work in comprehending PICS within the Colombian context and, in general, the Spanish speaking context.

In conclusion, the psychometric properties of the HABC-M instrument in its Spanish version demonstrate good validity and excellent reliability, which permits its use to detect and monitor patients with PICS.

Limitations. The stability coefficient through test-retest was not determined, given that it was not possible to program with the patients repetition of the scale’s application, besides travel difficulties for the survivors and researchers. The severity of the critical diseases of the survivors included in this study, evaluated through the APACHE II scale, was considered high, which is why the results may not be extrapolatable to survivors from other ICUs of lower complexity.

Recommendations. The HABC-M is a scale to detect the PICS; it should not be considered a diagnostic tool. Its results must be interpreted within the context of the clinical history and current state of health. The high scores are correlated with greater severity of the PICS.
